# Impact of catheter ablation versus medical therapy on cognitive function in atrial fibrillation: a systematic review

**DOI:** 10.1007/s10840-022-01196-y

**Published:** 2022-04-05

**Authors:** Neil Bodagh, Reuben Yap, Irum Kotadia, Iain Sim, Ajay Bhalla, Peter Somerville, Mark O’Neill, Steven E. Williams

**Affiliations:** 1 King’s College London, St Thomas’ Hospital, Westminster Bridge Road, London, SE1 7EH UK; 2 Princess Royal University Hospital, Orpington, UK; 3 Guys and St. Thomas’ NHS Foundation Trust, London, UK; 4 Centre for Cardiovascular Science, University of Edinburgh, Edinburgh, UK

**Keywords:** Atrial fibrillation, Catheter ablation, Cognitive dysfunction, Cognition, Dementia

## Abstract

**Purpose:**

Atrial fibrillation is associated with an increased risk of cognitive impairment. It is unclear whether the restoration of sinus rhythm with catheter ablation may modify this risk. We conducted a systematic review of studies comparing cognitive outcomes following catheter ablation with medical therapy (rate and/or rhythm control) in atrial fibrillation.

**Methods:**

Searches were performed on the following databases from their inception to 17 October 2021: PubMed, OVID Medline, Embase and Cochrane Library. The inclusion criteria comprised studies comparing catheter ablation against medical therapy (rate and/or rhythm control in conjunction with anticoagulation where appropriate) which included cognitive assessment and/or a diagnosis of dementia as an outcome.

**Results:**

A total of 599 records were screened. Ten studies including 15,886 patients treated with catheter ablation and 42,684 patients treated with medical therapy were included. Studies which compared the impact of catheter ablation versus medical therapy on quantitative assessments of cognitive function yielded conflicting results. In studies, examining new onset dementia during follow-up, catheter ablation was associated with a lower risk of subsequent dementia diagnosis compared to medical therapy (hazard ratio: 0.60 (95% confidence interval 0.42–0.88, *p* < 0.05)).

**Conclusion:**

The accumulating evidence linking atrial fibrillation with cognitive impairment warrants the design of atrial fibrillation treatment strategies aimed at minimising cognitive decline. However, the impact of catheter ablation and atrial fibrillation medical therapy on cognitive decline is currently uncertain. Future studies investigating atrial fibrillation treatment strategies should include cognitive outcomes as important clinical endpoints.

**Supplementary Information:**

The online version contains supplementary material available at 10.1007/s10840-022-01196-y.

## Introduction

The prevalence of atrial fibrillation increases with age and atrial fibrillation results in an increased risk of morbidity and mortality from stroke and congestive cardiac failure amongst other chronic diseases [1]. Improvements in life expectancy have contributed to an increased prevalence of dementia [2]. Although there is accumulating evidence of a causal link between atrial fibrillation and cognitive impairment [3], the mechanism of interaction is unknown.

Atrial fibrillation treatment is heterogenous comprising the use of rhythm control, rate control and anticoagulation therapy. The impact of these treatment strategies on cognition is unclear. Rhythm control with catheter ablation may be more successful at restoring and maintaining sinus rhythm than anti-arrhythmic drugs [4]. However, catheter ablation is also associated with an increased risk of cerebral emboli, particularly in the immediate post-ablation period [5]. Studies which have examined cognition before and after atrial fibrillation ablation have provided conflicting results [6–9]. We hypothesised that the risk of cognitive decline may differ in atrial fibrillation patients treated with catheter ablation compared with medical therapy.

The aim of this study was therefore to provide a systematic assessment of the effect of catheter ablation versus medical therapy on cognitive function. We present a systematic review of the available literature assessing cognitive function following catheter ablation compared to medical therapy (comprising rhythm and/or rate control in conjunction with anticoagulation where appropriate) for all patients with atrial fibrillation.

## Methods

This study was performed according to the Preferred Reporting Items for Systematic Reviews and Meta-Analyses (PRISMA) reporting guidelines [10].

### Data sources and searches

The PubMed, Ovid MEDLINE, Embase and Cochrane Library databases were searched from their inception to 17 October 2021. Reference lists of included articles were examined for additional studies. The search terms utilised were as follows: “atrial fibrillation” or “a fib” or “afib” or “AF” AND “cognition” or “cognitive impairment” or “cognitive” or “$dementia” or “$alzheimer” or “lewy” or “memory” or “vascular dementia” or “frontotemporal lobar” AND “catheter ablation” or “rfa” or “radiofrequency ablation” or “ablation” or “ca” or “cryoablation” or “cryoballoon” or “pulmonary vein.”

### Study selection and outcomes

Studies were included according to the following criteria: (1) studies which assessed cognitive function; (2) studies which included a diagnosis of dementia and/or its subtypes as an outcome; (3) studies which included both a group of atrial fibrillation patients treated with catheter ablation and patients treated with medical therapy (rate and/or rhythm control in conjunction with anticoagulation where appropriate). The following exclusion criteria were applied: (1) studies which did not include a group of patients treated with catheter ablation; (2) studies which did not include a group of patients treated with medical therapy; (3) case reports, editorials, reviews, conference proceedings and guidelines were excluded.

References were obtained and uploaded to Rayyan software [11]. Duplicate articles were removed. Two independent reviewers (NB and RY) screened the titles and abstracts of the studies. Articles considered relevant were advanced to full text review. Disagreements were resolved by consensus decision amongst the two authors. It was planned for a third reviewer (SEW) to arbitrate if disagreements could not be resolved; however, this was not required.

Outcomes assessed included diagnosis of dementia and its subtypes. These were ascertained according to International Classification of Disease coding data. Changes in cognitive function were assessed. We categorised these changes as (1) changes ≤ 3 months after therapy and (2) changes > 3 months after therapy.

### Data extraction and quality assessment

The following data from eligible studies were extracted (Table 1): PubMed ID, first author, year, country, follow up duration, study design, outcome measures, cognitive impairment ascertainment method, total number of study participants, number of patients who received catheter ablation, ablation procedure details, number of patients treated with medical therapy and details about the type of medical therapy used. Hazard ratios from propensity matched groups were used where available.

**Table 1:** Summary characteristics of the 10 studies included in the systematic review

PubMed ID	Year	First author	Title	Study design	Country	Length of follow up	Outcome measures	Method of cognitive impairment ascertainment	Total number of patients included in the study	Number with atrial fibrillation who received catheter ablation	Ablation procedure details	Number with atrial fibrillation treated with medical therapy	Medical therapy group details
30397930	2021	Wang	Radiofrequency and cryoballoon ablation improve cognitive function in patients with atrial fibrillation	Case–control	China	12 months	Telephone interview for cognitive status-modified (TICS-m) scores at baseline, 3 months and 12 months	Telephone interview for cognitive status-modified (TICS-m) scores ≤ 27 were defined as dementia and 28–31 were defined as mild cognitive impairment	139	98	Of the 98 patients treated with catheter ablation, 68 underwent radiofrequency catheter ablation with an irrigated contact force-sensing catheter whilst 30 underwent cryoballoon ablation using a double-walled balloon	41	The medical therapy group comprised patients who received conventional antiarrhythmic drug therapy. Patients in this group received antiarrhythmic drugs to lower ventricular rate below 110 beats/min and anticoagulant therapy according to CHA_2_DS_2_-VASc score Of the 41 patients treated with medical therapy, 21/41 (51.22%) were being treated with anticoagulant therapy
34261448	2021	Zhang	Incidence and risk factors of post-operative cognitive decline after ablation for atrial fibrillation	Case–control	China	6 months	Post operative cognitive decline at 48 h after ablation	Used a battery of 9 neuropsychological tests based on the Canadian Study of Health and Aging: (1) Consortium to establish a registry for Alzheimer’s disease (CERAD) auditory-verbal immediate, (2) CERAD auditory-verbal delayed, (3) & (4) trail making tasks A and B, (5) digit symbol substitution, (6) controlled oral word association, (7) CERAD semantic fluency, (8) and (9) grooved pegboard test-dominant hand and non-dominant hand Post operative cognitive decline was defined in an individual when the reliable change index score was less than − 1.96 on 2 tests and/or the combined z-score was less than − 1.96	287	190	Atrial fibrillation ablation procedures were performed under conscious sedation. For patients with paroxysmal atrial fibrillation, circumferential pulmonary vein isolation was utilised. Patients with persistent atrial fibrillation were treated with bilateral circumferential pulmonary vein antrum isolation and three linear ablation sets	87	Data were not available regarding the proportions of patients on rate and/or rhythm control therapy The medical therapy group comprised a similar proportion of patients taking oral anticoagulant therapy compared with the ablation group
31884008	2020	Hsieh	Catheter ablation of atrial fibrillation reduces the risk of dementia and hospitalization during a very long-term follow-up	Cohort	Taiwan	Mean follow up: 9.0 years	1. New onset of non-vascular dementia 2. Atrial fibrillation related hospitalisation	New onset of dementia of each subject was identified by International classification of disease (ICD-9-CM) codes	2344	787	Details about the catheter ablation procedure were not provided	787	In the group of patients treated with medical therapy, 459/787 (58.3%) were on anti-arrhythmic agents compared with 759/787 (96.4%) who underwent catheter ablation Warfarin was used in 448/787 (56.9%) of patients treated with medical therapy compared with 291/787 (37%) of patients who underwent catheter ablation
33022705	2020	Kim	Less dementia after catheter ablation for atrial fibrillation: a nationwide cohort study	Cohort	Korea	Median follow up: 52 months	1. Dementia 2. Dementia subtypes, including Alzheimer’s disease and vascular dementia	Diagnosis of dementia was defined using International classification of disease (ICD-10) codes	27097	9119	Details about the catheter ablation procedure were not provided	17978	Comparisons were made amongst groups propensity matched for anti-arrhythmic drug use and rate control therapy (comprising beta blocker, non-dihydropyridine calcium channel blockers and digoxin). The groups were also propensity matched for anticoagulant use (warfarin, non-vitamin K antagonist oral anticoagulant)
31299299	2020	Bunch	Stroke and dementia risk in patients with and without atrial fibrillation and carotid arterial disease	Cohort	USA	Mean follow up in atrial fibrillation, non-ablation group: 1459 days. Mean follow up in the atrial fibrillation, ablation group 2093 days	1. Long term dementia 2. 3-year dementia 3. 5-year dementia, 3-year stroke/transient ischaemic attack, 5-year stroke/transient ischaemic attack, long term stroke/transient ischaemic attack	Dementia was diagnosed using International classification of disease (ICD-9 and ICD-10) codes	11572	450	Details about the catheter ablation procedure were not provided	5336	Details about amiodarone, beta blocker, calcium channel blocker and anticoagulant therapy use are provided in supplementary table 2 of the article. Lower proportions of patients were treated with amiodarone (15.9% compared with 22.7%), beta blockers (46.8% compared with 60.9%), calcium channel blockers (32.4% compared with 36.7%) and anticoagulant therapy (34.7% compared with 56.0%) in the medical therapy arm
31624507	2019	Hyogo	One-year clinical outcomes of anticoagulation therapy among Japanese patients with atrial fibrillation: The Hyogo AF Network (HAF-NET) Registry	Cohort	Japan	Mean follow up: 355 days	1. Cerebral infarctions 2. Composite of new onset dementia, cardiac event requiring hospitalisation and all cause death	Mini‐Mental State Examination and/or Hasegawa dementia rating scales were used to define a diagnosis of dementia. Cut-off scores not mentioned	2113	614	Details about the catheter ablation procedure were not provided	1499	Details about the proportions of patients receiving rate and/or rhythm control therapy were not provided In total, 1842/2113 (87%) of the study participants were treated with anticoagulation therapy. In the catheter ablation arm, 439/614 (71.5%) patients were treated with anticoagulation therapy compared with 1403/1499 (93.6%) in the medical therapy group
31442075	2019	Jin	Atrial fibrillation catheter ablation improves 1-year follow-up cognitive function, especially in patients with impaired cognitive function	Case–control	Korea	1 year	Montreal cognitive assessment score at 3 months and 1 year	Used Reliable Change Index adjusted for practice effects to measure cognitive changes as described by Chelune et al. (1993). Cognitive improvement defined as a Reliable Change Index > 1.645 and deterioration as a Reliable Change Index ≤ 1.645	358	308	An open-irrigated tip catheter was used to deliver radiofrequency energy for ablation. Patients underwent pulmonary vein isolation and bidirectional block of the cavotricuspid isthmus. For patients with persistent atrial fibrillation, a roofline, posterior inferior line and anterior line were added	50	Details about the proportions of patients receiving rate and/or rhythm control therapy were not provided Details about anticoagulation therapy use were not provided
31915546	2019	Tischer	Prevalence and progression of cognitive impairment in atrial fibrillation patients after treatment with catheter ablation or drug therapy	Case–control study with patients recruited from 2 × randomised control trials	Germany	16.8 (11) months	Montreal cognitive assessment score and Mini mental state examination	Participants were classified as cognitively impaired if their cognitive function test scores were < 24 by Montreal cognitive assessment and/or < 27 by Mini mental state examination	46	18	Details about the catheter ablation procedure were not provided	28	Details about the proportions of patients receiving rate and/or rhythm control therapy were not provided Details about anticoagulation therapy use were not provided
23684686	2013	Medi	Subtle post-procedural cognitive dysfunction after atrial fibrillation ablation	Case–control	Australia	90 days after the procedure	Post operative cognitive decline at 2 days and 90 days	Assessed cognitive function using 8 neuropsychological tests, mini mental state examination, National Adult Reading Test and visual analogue scales. Post operative cognitive decline was identified using the reliable change index [14]. Post operative cognitive decline was defined in an individual when the reliable change index score was less than − 1.96 on 2 tests and/or the combined z-score was less than − 1.96	150	90	Patients with paroxysmal atrial fibrillation had an ablation strategy consisting of wide encirclement of the pulmonary vein antra. Patients with persistent atrial fibrillation had adjunctive ablation at the discretion of the treating electrophysiologist	30	Table 2 of the study provides details about the proportions of patients treated with calcium channel blockers, beta blockers, antiarrhythmic drug therapy and warfarin. The group of patients treated with medical therapy comprised 15/30 (50%) who had been treated with antiarrhythmic drug therapy and 9/30 (30%) of patients had been treated with warfarin
1410581	2011	Bunch	Patients treated with catheter ablation for atrial fibrillation have long-term rates of death, stroke, and dementia similar to patients without atrial fibrillation	Cohort	USA	Mean follow up in atrial fibrillation, no ablation group: 5.1 years. Mean follow up in atrial fibrillation, ablation group: 3.1 years	1. Total mortality, 1 year mortality, 3-year mortality, long-term mortality 2. Heart failure (1, 3 year long term) 3. Cerebrovascular accident (1, 3-year long term) 4. Dementia (1, 3-year long term) 5. Alzheimer’s (1, 3-year long term) 6. Senile dementia (1, 3-year long term) 7. Vascular dementia (1, 3-year long term)	International classification of disease codes were used to determine the diagnosis and subtype dementia into Alzheimer's disease, vascular dementia, senile dementia and non-specified dementia	37908	4212	Details about the catheter ablation procedure were not provided	16848	Details about the proportions of patients receiving rate and/or rhythm control therapy were not provided Details about anticoagulation therapy use were not provided

Quality assessment was performed using the Newcastle Ottawa Scale for cohort studies and a modified version for case–control studies. Three broad domains were evaluated including the selection of study groups, the comparability of these groups and the ascertainment of the exposure or assessment of outcome. An adequate follow up period was defined as 1 year for long-term cognitive function, and key control factors included stroke followed by age, gender and smoking history.

### Statistical analysis

Statistical analyses were performed using Review Manager version 5.4.1. The individual studies included in the meta-analysis used Cox proportional hazard regression models to determine hazard ratios. Where meta-analysis was performed, heterogeneity was assessed, and an *I*^2^ value ≥ 50% was considered significant. In this instance, a random-effects model was used to provide a more conservative estimate and because it is less influenced by the weighting of each study [12]. The inverse variance method based on a random-effects model was used to quantitatively summarise the outcome results and derive a pooled hazard ratio for dementia incidence in patients treated with catheter ablation versus medical therapy. A plan was made to assess for publication bias using visual inspection of funnel plot asymmetry if a minimum of 10 studies were included in the meta-analysis [13].

## Results

### Screening

**Fig. 1:**
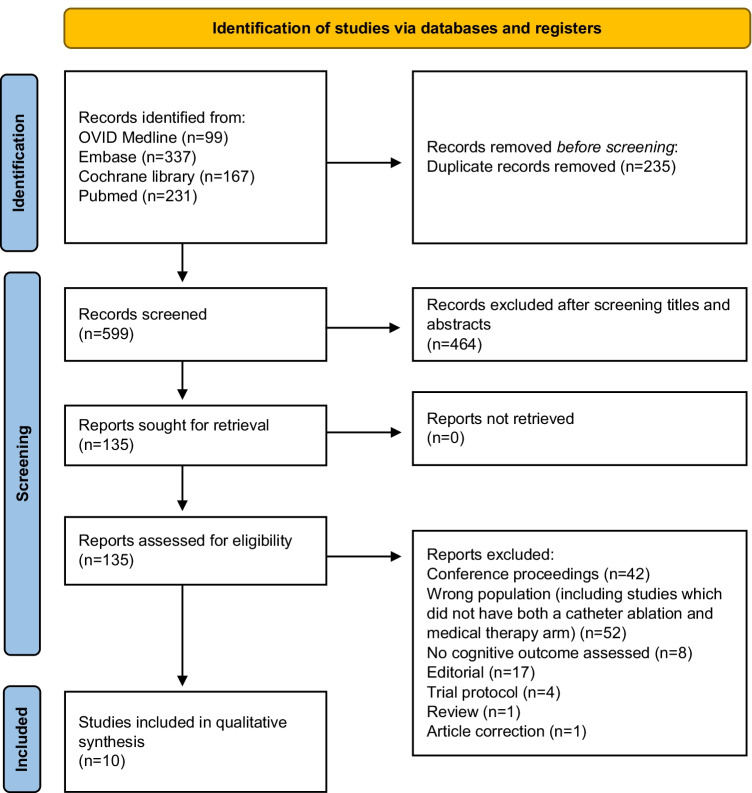
Preferred Reporting Items for Systematic Reviews and Meta-Analyses (PRISMA) flow diagram

The search strategy yielded 599 studies after duplicate removal. Full-text screening was performed on 135 studies which identified 10 studies meeting the inclusion/exclusion criteria. The Preferred Reporting Items for Systematic Reviews and Meta-Analyses flow diagram is shown in Fig. 1.

### Included studies

In the 10 included studies, 15,886 patients were treated with catheter ablation, and 42,684 patients were treated with medical therapy for atrial fibrillation. Detailed characteristics of the studies are provided in Table 1. Baseline characteristics of the study participants are detailed in supplementary Table S1.

### Assessment of study quality

The 10 included studies comprised 6 cohort studies, 4 case–control studies and no randomised control trials. Newcastle–Ottawa Scale scores for the included studies are depicted in Table 2. The study scores ranged from 6/9–9/9. The cohort studies [14–19] presented suitable cohort comparability and outcome assessments, though often failed to demonstrate that the outcome of interest (dementia or impaired cognitive function) was not present at the start of the study. Jin et al. fulfilled all domains appropriately [20]. Medi et al. and Zhang et al. lacked the comparability of other studies due to their methods of recruiting consecutive patients awaiting catheter ablation [21, 22]. These studies were consequently unable to control for key control factors (stroke, age, gender and smoking history).

**Table 2:** Newcastle–Ottawa Scale scores for (a) cohort studies and (b) case–control studies

A									
	Selection				Comparability	Outcome			
First author, year	Representation of the exposed cohort	Selection of the non-exposed cohort	Ascertainment of exposure	Outcome of interest was not present at the start of study	Comparability of cohorts on the basis of the design or analysis	Assessment of outcome	Was follow-up long enough for outcomes to occur	Adequacy of follow up of cohorts	Total
Wang, (2021)	✶	✶	✶	✶	✶	✶	✶	✶	8
Hsieh, (2020)	✶	✶	✶	✶	✶	✶	✶	✶	8
Kim, (2020)	✶	✶	✶		✶✶	✶	✶	✶	8
Bunch, (2020)	✶	✶	✶		✶✶	✶	✶	✶	8
Bunch, (2011)	✶	✶	✶		✶✶	✶	✶	✶	8
Hyogo, (2019)	✶	✶	✶		✶✶	✶	✶	✶	8
B									
	Selection				Comparability	Exposure			
First author, year	Case definition	Representativeness of the cases	Selection of controls	Definition of controls	Comparability of cases and controls on the basis of the design	Ascertainment of exposure	Same method of ascertainment for cases and controls	Non response rate	Total
Zhang (2021)	✶	✶	✶	✶			✶	✶	6
Jin (2019)	✶	✶	✶	✶	✶✶	✶	✶	✶	9
Tischer (2019)	✶	✶	✶	✶	✶	✶	✶	✶	8
Medi (2013)	✶	✶	✶	✶			✶	✶	6

### Cognitive function assessment

Numerous neuropsychological tests were utilised to assess cognitive function. These included mini mental state examination, Montreal cognitive assessment, Hasegwa dementia rating scale, national adult reading test, the visual analogue scale, the telephone interview for cognitive status-modified test and a well-established battery of 9 neuropsychological tests based on the Canadian Study of Health and Aging [23] (Table 1). Reliable change indices, as described by Chelune et al. [24] and Rasmussen et al. [25], were used to track changes in individuals’ test scores and assess for post ablation cognitive decline where reported.

### New-onset dementia

**Fig. 2:**
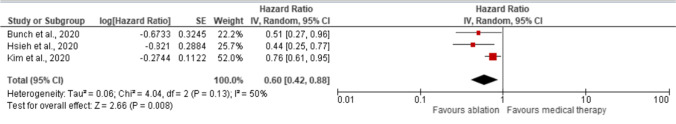
Forest plot demonstrating the results of the pooled meta-analysis assessing the hazard ratios for the development of dementia. Tests for heterogeneity were also performed using the *I*^2^ test

Of the 10 studies included, 4 reported hazard ratios for dementia incidence [14–17] according to International Classification of Disease coding data. Due to the small number of included studies, we did not test for publication bias. The duration of follow-up varied amongst the studies (Table 1). These studies demonstrated an association between patients treated with catheter ablation therapy and a lower incidence of dementia. The 95% confidence intervals were available for 3 of these studies (Fig. 2). A pooled meta-analysis of these studies demonstrated a hazard ratio of 0.60 (95% confidence interval 0.42–0.88, *p* < 0.05 with an *I*^2^ value of 50%). Hyogo et al. found that new onset dementia occurred in 8 of 2113 (0.4%) atrial fibrillation patients [18]. In the 614 patients who had been treated with catheter ablation, 3 (0.5%) had developed dementia over the 1 year follow up period. A hazard ratio was not obtained (potentially due to the low event rate). For the studies reporting numbers of patients who developed dementia, these data are summarised in supplementary Table S2.

### Dementia subtypes

Two studies reported incidence of dementia subtypes: Alzheimer’s disease and vascular dementia [15, 17]. Both studies reported that catheter ablation was associated with lower rates of Alzheimer’s disease compared with medical therapy. (Bunch et al. — hazard ratio 0.33, Kim et al. — hazard ratio 0.77). Kim et al. reported that catheter ablation was associated with a lower incidence of vascular dementia (hazard ratio 0.50 (95% confidence interval 0.33–0.74), *p* < 0.001). This contrasted to Bunch et al., who did not report a statistically significant difference (hazard ratio 0.74, (95% confidence interval not available), *p* = 0.37).

### Changes in cognitive function > 3 months after catheter ablation or medical therapy

Tischer et al. performed cognitive function assessments on patients in 1 centre recruited from the Catheter Ablation Versus Anti-arrhythmic Drug Therapy for Atrial Fibrillation Trial (CABANA) and Catheter Ablation versus Standard Conventional Therapy in Patients with Left Ventricular Dysfunction and Atrial Fibrillation (CASTLE AF) trials [26]. In these trials, patients with atrial fibrillation were randomly assigned to receive either catheter ablation or medical therapy. The group enrolled patients 12 months after trial randomisation and performed 2 cognitive function assessments at 6-month intervals to assess the impact of the aforementioned interventions. The study found no statistically significant changes in mini mental state examination or Montreal cognitive assessment scores in patients treated with catheter ablation or medical therapy. On the contrary, Jin et al. found that Montreal cognitive assessment scores improved 1 year post radiofrequency catheter ablation in a group of patients treated with catheter ablation (score at baseline: 25.4 ± 2.4, score after 1 year: 26.5 ± 2.3; *p* < 0.001), but not in a propensity-matched control group (score at baseline: 25.4 ± 2.5, score after 1 year: 24.8 ± 2.5; *p* = 0.012) [20]. Wang et al. used the telephone interview for cognitive status–modified test to assess cognitive function and found that scores improved in the catheter ablation arm (score at baseline: 36.74 ± 3.097, score at 1 year 39.56 ± 3.198) but not in the medical therapy arm (score at baseline: 36.41 ± 3.033, score at 1 year 34.44 ± 3.271) [19].

### Changes in cognitive function ≤ 3 months after catheter ablation or medical therapy

Three studies reported outcomes ≤ 3 months after catheter ablation [20–22]. Medi et al. and Zhang et al. tested cognitive function 48 h after ablation [21, 22]. In the study by Medi et al., patients had their procedure performed under general anaesthesia whereas the patients in the study by Zhang et al. had their procedure under conscious sedation. Medi et al. found that post ablation cognitive decline occurred in 17 of 60 patients with paroxysmal atrial fibrillation (28%; 95% confidence interval 18% to 41%), 8 of 30 patients with persistent atrial fibrillation (27%; 95% confidence interval 13% to 44%) and 0 of 30 control patients at 48 h (*p* = 0.007). Zhang et al. assessed for post ablation cognitive decline 48 h after ablation using 9 tests, based on the Canadian study of health and aging [23]. The authors found that post ablation cognitive decline occurred in 26 out of 190 patients (13.7%) treated with catheter ablation for atrial fibrillation.

Jin et al. and Medi et al. assessed cognitive function at 3 months. The study by Jin et al. found that baseline Montreal cognitive assessment score improved from 25.36 ± 2.39 to 26.57 ± 2.29 (*p* < 0.001) after 3 months in a propensity matched ablation group (*n* = 150). The control group’s Montreal cognitive assessment scores were 25.39 ± 2.53 at baseline and 25.24 ± 2.31 after 3 months (*p* > 0.05). Medi et al. found that post ablation cognitive decline at 90 days occurred in 8 of 60 patients paroxysmal atrial fibrillation patients treated with catheter ablation (13%; 95% confidence interval: 6 to 24%), 6 of 30 persistent atrial fibrillation patients treated with catheter ablation (20%; 95% confidence interval: 9 to 37%) and 0 of 30 control patients with atrial fibrillation (*p* < 0.03).

## Discussion

The major findings from this study were (1) there is a significant lack of randomised control trial evidence to assess the impact of catheter ablation versus medical therapy on cognitive outcomes in atrial fibrillation; (2) the impact of catheter ablation on the rate of cognitive decline in patients with and without pre-existing cognitive impairment is currently unknown; (3) the data currently available may suggest that patients treated with catheter ablation may have a lower risk of developing dementia; (4) the data regarding cognitive function in the immediate post ablation period are inconsistent necessitating further study to ascertain the short to medium term effects of catheter ablation on cognitive function.

### Risk of dementia in catheter ablation versus medical therapy

The studies which examined the incidence of dementia all found catheter ablation may be associated with a lower risk of developing dementia during follow-up compared with medical therapy [14–17]. The studies varied in their follow up durations with Hsieh et al., demonstrating that this pattern persisted in patients with a follow up period of 9.0 years. These findings were consistent across populations studied in 3 different countries. It is important to interpret these findings with caution. Whilst attempts to correct for underlying baseline differences were made through propensity matching, it is possible that unmeasured differences amongst the groups may have contributed to the findings. Patients referred for catheter ablation may have been less likely to develop dementia for reasons other than the treatment modality itself. We therefore cannot exclude the possibility of a selection bias favouring catheter ablation. The answer to the question of whether catheter ablation can prevent the onset of dementia in atrial fibrillation will come in the form of a randomised controlled trial.

### The link between catheter ablation and cognitive decline

The mechanistic link between catheter ablation and cognitive decline remains to be elucidated. The resolution of cerebral hypoperfusion with sinus rhythm restoration through catheter ablation may prevent cognitive decline [27]. Interestingly, Jin et al. found that patients with sustained atrial fibrillation after catheter ablation had lower improvements in cognitive function at 1 year compared with patients who maintained sinus rhythm [20] suggesting that sinus rhythm maintenance may attenuate cognitive decline. A study by Piccini et al. performed in patients treated with catheter ablation for atrial fibrillation found that patients with recurrent atrial tachycardia and/or atrial fibrillation after ablation had similar improvements in Montreal cognitive assessment scores after 3 months compared with those who did not [28]. This study was performed in patients recruited from the AXAFA-AFNET 5 trial, a study comparing continuous apixaban therapy to vitamin K antagonist therapy during ablation [8]. Unfortunately, these studies do not precisely quantify atrial fibrillation burden throughout their follow up periods. Studies which attempt to correlate cognitive function with duration in sinus rhythm will need to be performed over longer time periods. This could potentially be done with implantable and/or wearable technologies to quantify atrial fibrillation burden.

If sinus rhythm-induced improvement in cerebral perfusion was indeed the mechanism by which catheter ablation halted neurocognitive decline, then one could speculate that any rhythm control therapy would result in improvements in cognitive function. However, studies which have compared rhythm control therapy to rate control have provided conflicting results [29–31]. One plausible explanation for this discrepancy may be the variable efficacy of anti-arrhythmic medications to restore and maintain sinus rhythm. The effects of anti-arrhythmic medications and chemical cardioversion on cerebral perfusion patterns are currently unclear presenting an area for further study.

Verification of the precise interaction between atrial fibrillation rhythm control strategies and cognitive decline may enable tailored treatment towards subtypes of dementia. Our study showed that catheter ablation might be associated with a lower risk of Alzheimer’s disease. Chronic cerebral hypoperfusion is thought to exacerbate amyloid-beta neuropathology, through the upregulation of amyloid-beta producing enzymes and lowering of amyloid-beta clearing proteins [32]. Catheter ablation-induced resolution of hypoperfusion could explain this association. Intriguingly, the risk of vascular dementia was not lessened to the same extent. This may result from the association between catheter ablation and subclinical cerebral emboli [5].

The association of catheter ablation with asymptomatic cerebral infarcts has led to speculation that the procedure may increase the risk of cognitive impairment. The clinical significance of post ablation infarcts is unclear at present [33]. Asymptomatic cerebral infarcts have been related to an increased risk of dementia in the general population [34]. Whether this is true for the cerebral emboli which result from catheter ablation is unclear. Further studies are warranted to examine the impact of post ablation asymptomatic cerebral infarcts on cognitive function in the longer term.

### Changes in cognitive function > 3 months after ablation

Three studies performed repeated cognitive function assessments at intervals over specified time periods [19, 20, 26]. Tischer et al. recruited patients from the Catheter Ablation Versus Anti-arrhythmic Drug Therapy for Atrial Fibrillation Trial (CABANA) and Catheter Ablation versus Standard Conventional Therapy in Patients with Left Ventricular Dysfunction and Atrial Fibrillation (CASTLE AF) studies. Patients were assessed at least 12 months after randomisation to catheter ablation or medical therapy and cognitive tests performed at intervals of 6 months. The authors found no statistically significant changes in cognitive function. Jin et al. demonstrated that Montreal cognitive assessment scores improved 12 months after catheter ablation. These findings were corroborated by Wang et al., who found improvements in telephone interview for cognitive status-modified test scores at 12 months for patients treated with catheter ablation but not medical therapy. This discrepancy may be explained by the fact that patients recruited by Tischer et al. were older with a higher prevalence of pre-existing cognitive impairment compared with Wang et al. and Jin et al. Furthermore, cognitive decline is often a gradual process. The time periods between serial cognitive function testing differed amongst the studies and could have contributed to the differing results.

### Cognitive function ≤ 3 months after ablation

The association between catheter ablation and subclinical cerebral emboli [5] has led to speculation that catheter ablation may worsen cognitive function. A notable finding from our study was that Jin et al. found that catheter ablation was associated with cognitive assessment score improvement after 3 months [20] whereas Medi et al. found a worsening of cognitive function at this time period [21]. The studies differed in the type of ablation catheter utilised, their method of anaesthesia and anticoagulation strategies. Differences in method of anaesthesia may also contribute to the difference in post ablation cognitive decline incidence at 2 days in the studies by Zhang et al. and Medi et al. [21, 22]. A lower proportion of patients in the study by Zhang et al. had their ablation under general anaesthesia compared with Medi et al. It is becomingly increasingly recognised that the procedural protocols and ablation tools used have an influence on the prevalence of silent cerebral infarcts [5]. Study heterogeneity is therefore likely to have contributed significantly to the differences in these results.

### Limitations

The main limitation of our study was the lack of randomised control trial level evidence. This makes it difficult to make firm conclusions based on the data currently available. Questions pertaining to the impact of catheter ablation on cognitive function in patients with pre-existing cognitive impairment, risk of dementia and short- to medium-term cognitive function remain unanswered based on the evidence reviewed in this study. The lack of such data stresses the requirement for further study to investigate the impact of atrial fibrillation treatment modalities on cognitive function.

Selection bias may have contributed to the findings of this review. For example, variations in age, sex and cardiovascular comorbidities between groups treated with either medical therapy or catheter ablation in the available literature may have contributed to differences in cognitive outcomes between groups. Most of the studies identified were observational in nature. Whilst 7 of the 10 studies attempted to match for confounders, unaccounted-for confounders could have explained some of the results observed. Additionally, various assessments of cognition were used. Some studies used an International Classification of Disease diagnosis of dementia as an endpoint whereas others used cognitive function assessments. Given the heterogeneity of these cognitive assessment methods, it is possible that the type of assessment used may have influenced the results observed. Most of the outcomes discussed in this review are therefore reported descriptively. This limitation reflects the limitations inherent in the available published data, again highlighting the need for further mechanistic research in this area.

The medical management and types of anticoagulation strategies utilised also differed likely resulting in variability amongst the studies. The same can be said of the ablation procedures used. Many of the studies included in the systematic review failed to provide details about the medical management and/or catheter ablation strategies employed (Table 1). This is likely to have affected the results obtained and importantly precludes meaningful assessment of the influence of procedural characteristics and choice of ablation technology on cognitive outcomes. Furthermore, baseline cranial imaging was not performed in the studies included. We cannot exclude the possibility of pre-existing abnormalities affecting the results of the studies.

### Future directions

To mitigate the risk of selection bias, randomised trials are required to compare the effects of atrial fibrillation treatment strategies on cognition. The Cognitive Impairment in Atrial Fibrillation study (DIAL-F, ClinicalTrials.gov Identifier: NCT01816308) will compare catheter ablation against antiarrhythmic drug therapy. Future trials to assess the impact of other modalities such as electrical cardioversion and rate control would also be useful. Catheter ablation itself is also heterogeneous, and it is therefore feasible that different catheter ablation techniques will have differing effects on cognition presenting another important area for future study.

Studies should also be designed to investigate the mechanisms through which atrial fibrillation treatment strategies may affect cognition. The Neurocognition and Greater Maintenance of Sinus Rhythm in Atrial Fibrillation (NOGGIN AF, project number 1R01AG074185-01) trial will compare cognitive function in patients treated with catheter ablation versus medical therapy. This study will compare structural cortical characteristics and cerebral perfusion patterns providing important information about how such treatment strategies could differentially affect cerebral structure and function. Additionally, it would be useful to ascertain whether there is a relationship between atrial fibrillation treatment and levels of biomarkers indicative of cognitive impairment in plasma and cerebrospinal fluid.

Studies should also be designed to identify whether catheter ablation–induced changes in other parameters such as cardiac function and/or symptom burden correlate with changes in cognitive function. This could enable a greater understanding of the mechanistic link between atrial fibrillation and cognitive decline. It would also be useful to examine whether a patient’s atrial fibrillation classification influences the risk of cognitive decline with medical or interventional therapy. This information could be used to identify the patient groups most likely to benefit from atrial fibrillation treatment strategies aimed at halting cognitive decline.

## Conclusion

The link between atrial fibrillation and dementia is increasingly reported. Treatment strategies should be aimed at minimising the cognitive decline process observed in patients with atrial fibrillation. Given the limitations of the available data, we are unable to make firm conclusions; however, this study suggests that catheter ablation may offer promise to prevent neurocognitive decline in atrial fibrillation. Further studies are therefore warranted to elucidate the mechanistic link between atrial fibrillation and cognitive decline, delineate the true impact of catheter ablation versus medical therapy on cognitive function, and to strive to identify the patient groups most likely to benefit from treatment aimed at halting cognitive decline.

## Supplementary Information

Below is the link to the electronic supplementary material.

Supplementary file1 (DOCX 43 KB)
