# Supplementary material for: Impact of catheter ablation versus medical therapy on cognitive function in atrial fibrillation: a systematic review
Source: J Interv Card Electrophysiol. 2022 Apr 5;65(1):271–86. doi: 10.1007/s10840-022-01196-y (PMC9550702; doi:10.1007/s10840-022-01196-y)
Supplement: Supplementary file 1 — Supplementary file1 (DOCX 43 KB) [file 10840_2022_1196_MOESM1_ESM.docx]

SUPPLEMENTARY TABLE S1. Baseline characteristics of the patients included in the studies.

|  |  | **Atrial fibrillation with Catheter Ablation** | | | | | | **Atrial fibrillation without Catheter Ablation** | | | | | |
| --- | --- | --- | --- | --- | --- | --- | --- | --- | --- | --- | --- | --- | --- |
| **First Author, Year** | **Total number of patients** | **Number with atrial fibrillation who received catheter ablation** | **Mean Age (± standard deviation)** | **Male (%)** | **Paroxysmal atrial fibrillation (%)** | **CHA_2_DS_2-_VASC Score (± standard deviation)** | **Anticoagulant (Warfarin or Non Vitamin K antagonist oral anticoagulant %)** | **Number with atrial fibrillation treated with medical therapy** | **Mean Age (± standard deviation)** | **Male (%)** | **Paroxysmal atrial fibrillation (%)** | **CHA_2_DS_2-_VASC Score (± standard deviation)** | **Anticoagulant (Warfarin or Non Vitamin K antagonist oral anticoagulant %)** |
| Wang, 2021 | 139 | 98 | Data not provided | Data not provided | Data not provided | Data not provided | Data not provided | 41 | 64.51 ± 8.12 | 53.66 | 46.34 | 2 (0, 5) | 51.2 |
| Zhang, 2021 | 287 | 190 | 66.6 ± 5.4 | 59.5 | 68.9 | 40% - <2 males, <3 females | 61.1 | 97 | 67.7 ± 5.4 | 59.5 | 68.9 | 46.8% - <2 males, <3 females | 57.7 |
| Hsieh, 2020 | 2344 | 787 | 54.1 ± 11.5 | 70.1 | Data not provided | 1 | 56.9 | 787 | 54.9 ± 11.6 | 70.4 | Data not provided | 0 | 37 |
| Kim, 2020 | 27097 | 9119 | 57 | 76.5 | Data not provided | 2 | 64.8 | 17978 | 67 | 67.5 | Data not provided | 4 | 64.7 |
| Bunch, 2020 | 11572 | 450 | 73.7 ± 10.7 | 24.2 | Data not provided | 4.5 ± 1.9 | 56 | 5336 | 73.5 ± 12.2 | 17.3 | Data not provided | 4.5 ± 1.8 | 34.7 |
| Hyogo, 2019 | 2113 | 614 | 65.7 ± 10.3 | Data not provided | Data not provided | 2.23 ± 1.68 | 71.5 | 1499 | 71.8 ± 10.6 | Data not provided | Data not provided | 2.73 ± 1.5 | 93.6 |
| Nyun-Jin, 2019 | 358 | 308 | 60.6 ± 9.1 | 71.4 | 65.9 | 2 ± 1.6 | Data not provided | 50 | 60.3 ± 7.5 | 78 | 34% | 1.5 ± 1.3 | Data not provided |
| Tischer, 2019 | 46 | 18 | Data not provided | Data not provided | Data not provided | Data not provided | Data not provided | 28 | Data not provided | Data not provided | Data not provided | Data not provided | Data not provided |
| Medi, 2013 | 150 | 90 | Data not provided | Data not provided | Data not provided | Data not provided | Data not provided | 30 | 53 ± 9 | 77 | Data not provided | CHADs2 0.6 ± 0.8 | 30 |
| Bunch, 2011 | 37908 | 4212 | 64.8 ± 12.7 | 60.8 | Data not provided | Data not provided | Data not provided | 16848 | 66 ± 13.3 | 60.8 | Data not provided | Data not provided | Data not provided |

SUPPLEMENTARY TABLE S2. Numbers of patients who developed dementia in studies which reported the incidence of dementia. Matched groups were used where available.

|  | **Number of patients treated with catheter ablation** | **Number of patients diagnosed with dementia in ablation group** | **Number of patients treated with medical therapy (matched groups)** | **Number of patients diagnosed with dementia in medical therapy group** |
| --- | --- | --- | --- | --- |
| Bunch et al., 2020 | 442 | 23 | 5107 | 501 |
| Hsieh et al., 2020 | 787 | 29 | 787 | 55 |
| Kim et al., 2020 | 5863 | 164 | 5863 | 308 |
| Hyogo et al., 2019 | 614 | 3 | 1499 | 5 |
| Bunch et al., 2011 | 4212 | 24 | 16848 | 480 |
